# Refractory ascites after laparoscopic cholecystectomy: a case report

**DOI:** 10.1186/s12893-022-01758-x

**Published:** 2022-08-17

**Authors:** Xiaoyun Cheng, Jin Huang, Aiming Yang, Qiang Wang

**Affiliations:** 1 Department of Gastroenterology, Peking Union Medical College Hospital, Chinese Academy of Medical Sciences and Peking Union Medical College, Beijing, 100730 China; 2 Department of Internal Medicine, Peking Union Medical College Hospital, Chinese Academy of Medical Sciences and Peking Union Medical College, Beijing, 100730 China

**Keywords:** Refractory ascites, Chylous leakage, Laparoscopic cholecystectomy, Lymphatic vessels, Lymphoscintigraphy, Case report

## Abstract

**Background:**

Laparoscopic cholecystectomy is a common surgical option for gallstone disease with minimal trauma and rapid recovery. Ascites is a relatively uncommon complication after laparoscopic cholecystectomy and is more frequently observed in patients with preoperative abnormal liver function. However, patients without underlying liver disease develop refractory ascites after laparoscopic cholecystectomy are rare. We report a case of massive ascites caused by lymphatic injury after laparoscopic cholecystectomy.

**Case presentation:**

A 63-year-old woman complained of abdominal discomfort and distension at the twelfth day after a laparoscopic cholecystectomy for gallbladder stones. Subsequently, the patient developed spontaneous bacterial peritonitis and a decreased output of urine. Abdominal computed tomography (CT) identified abdominal effusion. The patient received abdominocentesis and the volume of slightly turbid yellow ascites averaged 1500–2000 ml per day. The results of laboratory analysis of ascitic fluid showed the following: serum-ascites albumin-gradient (SAAG), 11–12 g/L; albumin, 11–14 g/L; triglycerides, 0.91 mmol/L. After the diuretic therapy, repeated large-volume paracentesis with albumin supplementation, administration of antibiotics and renal vasodilating medications, the patient’s symptoms did not relieve. Lymphoscintigraphy found a small amount of radioactive filling in the abdominal cavity. The patient finally received surgery with detection and ligation of the lymphatic leak. The ascites disappeared and the patient recovered well.

**Conclusions:**

For patients with atypical characteristics of chylous ascites, lymphoscintigraphy could help to localize and qualify the diagnosis. Surgical treatment could be considered when conservative treatment fails.

## Background

Identifying the cause of ascites is essential in the evaluation of a ascites case [1]. Common causes of ascites include liver cirrhosis, portal hypertension, congestive heart failure, nephropathy, abdominal and pelvic tumors, et al. [2]. Refractory ascites caused by lymphatic fistula after laparoscopic cholecystectomy is rare in clinical practice [3]. In this study, we reported a case of a 63-year-old woman with refractory ascites after laparoscopic cholecystectomy for gallbladder stones.

## Case presentation

**Fig. 1:**
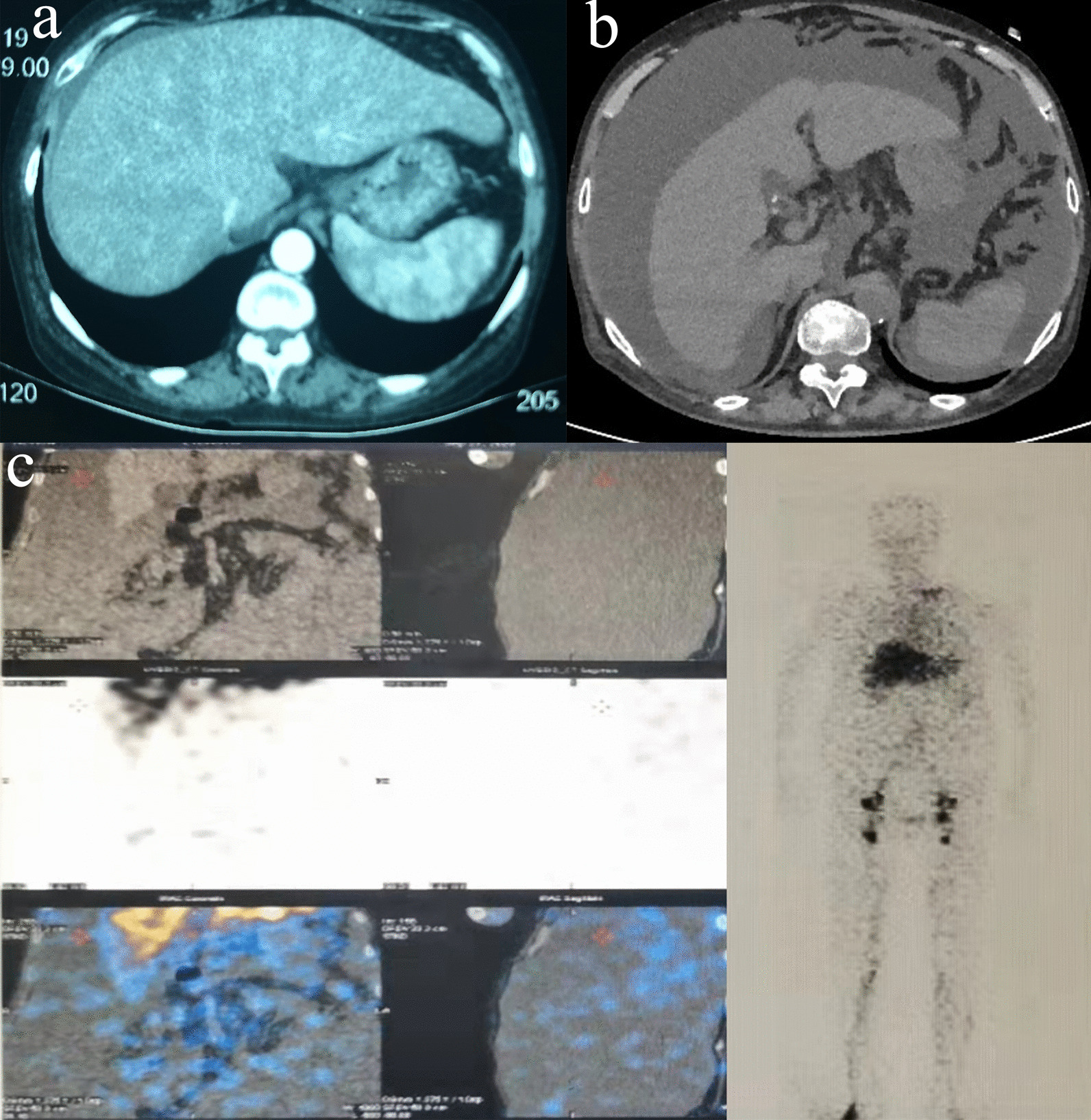
Imaging examinations. **a** Abdominal CT indicated a small amount of effusion around the liver before laparoscopic cholecystectomy; **b** Abdominal CT showed thickening of the peritoneum, a large amount of ascites, a decreased liver volume compared to pre-operation; **c** Lymphoscintigraphy revealed that a small amount of radioactive filling was found in the abdominal cavity

A 63-year-old woman was referred to our hospital with progressing abdominal distention for 3 months and oliguria for 2 months. The patient underwent laparoscopic cholecystectomy for gallbladder stones 3 months ago. Laboratory data before laparoscopic cholecystectomy was albumin, 32.7 g/L; prothrombin time 13.1 s. Preoperative abdominal computed tomography (CT) scan indicated a small amount of effusion around the liver (Fig. 1a) and no evidence of cirrhosis and splenomegaly was observed. Twelve days after surgery, the patient complained of abdominal discomfort and distension. Abdominal CT identified abdominal effusion. Then, the patient received abdominocentesis and the volume of slightly turbid yellow ascites averaged 1500–2000 ml per day. Gastroscopy revealed no esophageal and gastric varices. Endoscopic retrograde cholangiopancreatography (ERCP) revealed no biliary fistula. Positron emission tomography-computed tomography did not show any abnormal findings. After conservative treatment of diuretics, albumin administration, and repeated drainage for ascites, decreased urine volume, increased serum creatinine, and persistent ascites occurred. The patient was referred to our hospital with abdominal drainage tube for further clarifying the etiology.

The patient used to drink 300 mL red wine daily. She had taken an herbal weight loss product 10 years before. Systematic physical examination revealed a grossly distended abdomen with dilated collateral veins on the abdominal wall and severe bilateral lower extremity pitting edema. Shifting dullness was positive. Liver and spleen were untouched.

**Fig. 2:**
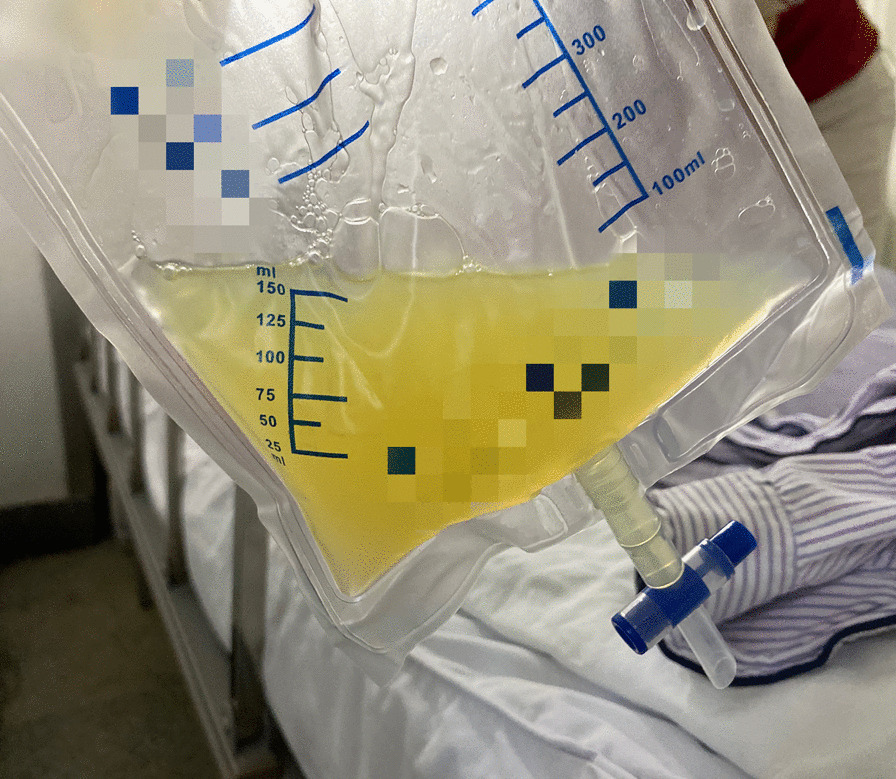
Ascites collected in the drain bag. The appearance of ascites was slightly turbid yellow fluid

Laboratory test results were as follows: white blood cell counts, 10,830 /μL; hemoglobin level, 13.4 g/dL; platelets, 364,000 /μL; albumin level, 25 g/L; alanine aminotransferase, 37 IU/L; total bilirubin, 6.4 μmol/L; creatinine, 171 μmol/L; urea, 24.39 mmol/L; triglycerides, 1.34 mmol/L; prothrombin time, 14–20 s; activated partial thromboplastin time, 30–47 s; fecal occult blood testing, negative; erythrocyte sedimentation rate, 4 mm/h; purified protein derivative, negative; T cell enzyme-linked immune-spot assay, negative; hepatitis B surface antigen, negative; hepatitis C virus antibody, negative; anti-nuclear antibody, negative; type IV collagen, 167 ng/ml; hyaluronic acid 888 ng/ml. Ascites analysis revealed that the gross appearance was straw-colored (Fig. 2), white blood cells was 3727 /μL (multinucleate cell, 89.7%), albumin was 11–14 g/L, serum-ascites albumin-gradient (SAAG) was 11–12 g/L, triglycerides was 0.91 mmol/L, total bilirubin and amylase were within normal limits. Ascites culture was positive for Enterococcus faecalis, Enterobacter cloacae, Acinetobacter pittii and Pseudomonas stutzeri. No fungi or Mycobacterium tuberculosis were present in the ascites culture. The cytological evaluation of the ascitic fluid showed no evidence of malignant cells.

Liver transient elastography demonstrated the liver stiffness was 53.1 kPa. Abdominal CT scan showed thickening of the peritoneum, a large amount of ascites, a decreased liver volume compared to pre-operation (Fig. 1b). Computed tomography angiography showed the main trunk of the portal vein was 14 mm wide at its widest point. No abnormal findings were observed by the lymphangiography. Lymphoscintigraphy found there was a small amount of radioactive filling in the abdominal cavity (Fig. 1c). Therefore, the patient was diagnosed with chyle leak following laparoscopic cholecystectomy.

We gave her simultaneous administration of human albumin solution, diuretics, and abdominal drain. After the ascitic fluid culture results turned negative, the volume of ascites did not decrease. Meanwhile, terlipressin and midodrine were administrated to the patient to improve renal function. Considering the possibility of the presence of chyle leak, the patient received laparotomy. During the operation, light yellow lymph fluid overflowed continually on the surface of the hepatoduodenal ligament. Methylene blue solution was injected into the subcapsule of the liver margin and was observed at the leakage of the lymphatic vessel of hepatoduodenal ligament. Then, ligation of abdominal lymphatic fistula was performed and the lymphatic fluid leakage was significantly reduced after suture.

After the operation, the patient's ascites disappeared, blood pressure stabilized, and urine output recovered. During the 12-month follow-up, she was in good condition.

## Discussion and conclusions

The etiology of ascites after surgery is variable. To clarify the cause of ascites plays a pivotal role in guiding the clinical treatment. A large amount of ascites after laparoscopic cholecystectomy is more common in patients with chronic liver disease. The mechanism may be the change of CO_2_ pneumoperitoneum pressure during the operation leads to ischemia–reperfusion injury in the liver, aggravating liver cells damage. In addition, the hyperdynamic circulatory state and abnormal structure of the lymphatic system caused by liver cirrhosis may be involved in the production of chylous ascites after upper abdominal surgery. Therefore, for patients with chronic liver disease, preoperative liver function evaluation is required. Patients with liver function of Child C need to be treated carefully. In our case, SAAG ≥ 11 g/L and ascites albumin < 25 g/L were highly indicative of portal hypertension related ascites due to cirrhosis. But the evidence for the diagnosis of liver cirrhosis was insufficient due to the normal liver metabolic function. Meanwhile, there was no imaging evidence of portal hypertension, such as portal vein widening, splenomegaly, and varicose veins. We hypothesized that on the basis of chronic liver damage, secondary portal hypertension factors probably involved in the continuous production of ascites.

Bile leakage, pancreatic leakage and chyle leakage are also common causes of ascites secondary to abdominal surgery. For this patient, ERCP ruled out the possibility of biliary duct injury, and the levels of bilirubin, amylase, and lipase of ascites were within the normal range. We found that the levels of albumin and triglycerides of ascites changed with the daily infused amount of albumin, and SAAG value was fluctuating during the treatment. Thereby, we considered the possibility of chyle leakage. Chylous ascites means lymph fluid enters the abdominal cavity, which is characterized as lipid rich milky or creamy fluid. The diagnosis of chylous ascites meets at least one of the following: the level of triglycerides in ascites > 1.25 mmol/L, positive results of lymphangiography or lymphoscintigraphy with the contrast agent entering the abdominal cavity. In this case, the appearance of ascites and the value of triglycerides did not meet the diagnostic criteria for chylous ascites. However, chylous ascites could not be completely ruled out due to low triglycerides level in ascites, as the large amount of ascites might dilute the concentration of ascites triglycerides. The lymphoscintigraphy indicated the existence of abdominal chyle leakage. Combined with performance seen during laparotomy, we speculated that the cause of chylous leakage might be a dissection conducted too close to the hepatoduodenal ligament or incomplete ligation during the laparoscopic cholecystectomy.

Chyle leakage caused by laparoscopic cholecystectomy is rare. The diagnosis needs to combine medical history, physical examination, ascites test, and imaging examination. Chylous ascites typically has a milky, cloudy, and turbid appearance. Ascites triglycerides > 1.25 mmol/L or > 2–8 times of blood triglycerides supports the diagnosis of chylous ascites. Abdominal ultrasound and CT scan contribute to locate and puncture ascites, while lymphoscintigraphy and lymphangiography are conductive to locate the lymphatic vessel damage for further surgical treatment. Treatments include medium-chain triglyceride diet, total parenteral nutrition, somatostatin analogs, and surgical treatment. In previous case reports [4–8], the symptom of abdominal discomfort usually occurred 1–14 days after laparoscopic cholecystectomy, and their ascites were all milky white turbid liquid. The ascites triglycerides were significantly increased (7.64–18.4 mmol/L). Chylous ascites relived by conservative treatment in 3 cases and the other 2 cases received surgical treatment.

Due to the atypical appearance and normal triglycerides of ascites, it is difficult to distinguish chyle leakage from portal hypertension related ascites and make the diagnosis. Typical chyle pleural or ascites fluid is white and opaque in appearance, about 20–50% of chyle fluid does not have a milky white turbid appearance observed by naked eyes [9]. So, visual inspection has poor diagnostic sensitivity for chyle fluid. In addition, the triglycerides cut-off value for diagnosing chylous ascites is still unclear. A study has reported that patients with ascites triglycerides < 1.25 mmol/L can also be identified as chylous ascites by lymphoscintigraphy. Lipoprotein electrophoresis is more reliable in the detection of chylomicrons [9].

In conclusion, refractory ascites caused by chyle leakage after laparoscopic cholecystectomy is a relatively rare postoperative complication. Combining medical history and examinations are important for early diagnosis. For patients with atypical characteristics of chylous ascites, lymphoscintigraphy or lymphangiography could help the localization and qualitative diagnosis. When conservative treatment fails, surgical treatment should be considered.

## Data Availability

Not applicable.

## Abbreviations

SAAG: Serum-ascites albumin-gradient
ERCP: Endoscopic retrograde cholangiopancreatography
CT: Computed Tomography
